# What Matters to Others: A High-Threshold Account of Joint Attention

**DOI:** 10.1007/s11245-024-10021-2

**Published:** 2024-04-17

**Authors:** Anna Bloom-Christen

**Affiliations:** Department of Anthropology, University of California, Los Angeles, Haines Hall; 375 Portola Plaza, Los Angeles, CA 90095 USA

**Keywords:** Joint attention, Anthropology, Participant observation, Participant attention, Perceptual common knowledge

## Abstract

If only implicitly, social anthropology has long incorporated joint attention as a research technique employed in what anthropologists call “the field”. This paper outlines the crucial role joint attention plays in anthropolgical fieldwork—specifically in Participant Observation—and advances the position that joint attention is a goal rather than a starting point of fieldwork practice. Exploring how anthropologists tentatively use attention as a methodological tool to understand other people’s lifeworlds, this paper draws parallels between Participant Observation and ordinary everyday interactions, thus teasing out a view of *joint* attention as a goal to be reached only by means of knowing what matters to others in the context of the lifeworld they inhabit.

## Introduction

I can attend to your stream of consciousness, just as I can attend to my own, and I can, therefore, become aware of what is going on in your mind. In the living intentionality of this experience, I “understand” you without necessarily paying any attention to the acts of understanding themselves. This is because, since I live in the same world as you, I live *in* the acts of understanding you.

— Alfred Schütz (1967, 140)

Joint attention plays a vital connective role in the social spaces of all societies. Its key functions include communication, social development, and joint action (Wilby 2023). Philosophers study attention in its solitary and shared form as a means to understand why humans think, feel, and act the way they do (Eilan et al. 2005; Posner 2011; Wu 2014). The same goes for anthropologists, albeit their concern with attention is not expressed in a heap of publications and topically designated research projects. While anthropology experiences a growing disciplinary imperative to “pay attention to attention” (Cook 2018) and has advanced proposals for how to study attention anthropologically (Pedersen et al. 2021), the systematic use of attention as a theoretical lens through which to understand human interaction is still in its beginnings. The main disciplinary concern has been the emic study of attention as it occurs in the lifeworlds of past and present cultures and societies. The research reflecting this trend asks versions of the question “How does attention manifest itself in particular cultural and social contexts?”.^1^

Meanwhile, attention has always been central to anthropological fieldwork, if in a somewhat hidden, taken-for-granted way. Recognized as a culturally coded phenomenon, *joint* attention is a perceptual objective of Participant Observation, the main method empirically employed by social anthropologists. Learning to follow their research partners’ gaze, anthropologists are uniquely fixated on joint attention as a form of joint action in that joint attention resembles an interim goal to be reached in order to make the sort of knowledge claims on which their forthcoming ethnographies are supposed to be based. The researcher’s task is to learn what matters to the people whose lifeworld she seeks to understand—loosely speaking, to see the world with their eyes—and a prime way to do that is to learn to pay attention to what they pay attention to as they are paying attention to it. In other words, the goal is joint attention.

This paper explores joint attention through the lens of the anthropologist plunging into ‘the field’. Figuratively following the gaze of those who seek to literally follow the gaze of others, joint attention will be considered as an interim goal to be reached by means of jointly acting with people in order to gain contextual knowledge about their lifeworlds. The aim is to characterize a demanding, high-threshold form of joint attention, one that thrives through a mutual understanding of the shared object of attention’s contextual meaning.

Section 2 introduces the role of Participant Observation in the disciplinary history of anthropology and places joint attention in the midst of its methodological toolkit.

Section 3 raises two challenges the research method faces, both of which are linked to the question of how demanding the conditions for joint attention should be in an anthropological context. The first challenge is epistemological and addresses the “problem of sharing the view ahead”. The second challenge targets moral implication of making knowledge claims about others by assuming a demanding notion of joint attention as a fulfilled goal.

Section 4 reconsiders what I call the perceptual timeline of Participant Observation by moving joint attention away from the beginning to the middle section of fieldwork practice. Here I will argue that joint attention is the result of participation, which in turn begins with attention as a form of distant observation. Drawing on empirical evidence from my own fieldwork in South Africa, I will offer a phenomenological account of joint attention inspired by Alfred Schütz’s observation that sharing a contextual frame of reference is to let go of the idea to pay attention to attention. Joint attention, in this view, is possible and actualizes as I “live in the act of understanding you” (Schütz 1967, 140).

The conclusion feeds those insights into the philosophcial discussion on what joint attention is. Taking cues from anthropology, this paper advances the position that joint attention should be studied not merely as a given everyday phenomenon, but also as an ideal that can only be partially fulfilled by continuous attentive participatory practice. Joint attention, as it is aimed at in anthropological fieldwork, will thus prove a valuable case to advance its characterization as an epistemologically demanding shared act of understanding one another.

## Joint Attention in Anthropological Fieldwork

Attention has long played an important if mostly tacit part in many anthropological debates and subfields, such as the ethnographic studies on rituals and religious experience (Novellino 2009; Pedersen et al. 2021). Although attention is an acknowledged crucial aspect of anthropological inquiry, its systematic use as a theoretical lens through which to understand human interaction is still in its beginnings.^2^

Meanwhile, part of the history of social anthropology could be coherently told in terms of how anthropologists came to agree that successful fieldwork depends on the degree to which joint attention can be achieved. For social anthropologists have gradually incorporated attention in their methodological toolkit. Baked into their principal method—Participant Observation—attention is a vital part of their long-term fieldwork practice. To spend time with one’s research partners means to participate in their everyday activities, and to gradually learn what to pay attention to by means of what I propose to call *participant attention*.

Here is a brief recap of the disciplinary history, which is fundamentally shaped by the development of its methods. Social anthropologists rely on qualitative empirical data derived from long term, on-site interactive research with the people they seek to study. To date, this kind of research is commonly known as ‘Participant Observation’.^3^ ‘Participant Observation’ is an umbrella term for a range of methods which conceptually overlap with ‘ethnography’ and ‘fieldwork’ while additionally specifying the manner of perceptual engagement (Spradley 1980; DeWalt & DeWalt 2002; Holt 2006).^4^ Textbooks define Participant Observation along the lines of an “engagement in social interaction between the researcher and informants in the milieu of the latter” (Taylor and Bodgan 1984, 15), thus “exploring the nature of particular social phenomena” (Atkinson and Hammersley 2007, 248). The *SAGE Handbook of Social Anthropology* proposes that conducting Participant Observation means to live “in a culture that is not your own while also keeping a detailed record of your observations” (2012, xii).

This has not always been so. Social anthropology started out and long remained committed to the idea that observation—that is, paying attention from a safe distance—is the best way to collect data. Part and parcel of the history of Western scientific thinking, observation is a cornerstone of the “scientific method” and a key feature of any empirical research where data is sought to be consistently and unobtrusively collected. Following this paradigm of scientific rigor bound up with the ideal of objectivity, paying attention from afar was the basis on which a first generation of anthropologists wrote their ethnographies (today perhaps more aptly located within the genre of travel literature, or perhaps ‘true adventure’). Attention was paid not together *with* the exoticised protagonists of these texts, but *to* them, typically filtered through the Western gaze of a white male. Stereotyped as either the noble or not-so-noble savage, ‘the other’ was being *watched* in what was considered their natural, self-contained habitat. Joint attention was neither a goal nor a focus, as the researcher alone was supposed to know what was worth observing.^5^

The turn towards participation happened gradually during the first half of the twentieth century (de Pina-Cabral 2018). Bronisław Malinowski is considered the main figure to whom the systematic definition and use of *Participant* Observation is attributed. In *Argonauts of the Western Pacific* (1961[1922]), Malinowski lays out his vision of how “to grasp the native’s point of view, his relation to life, to realize his vision of his world” (1961[1922], 25). Stepping off the observation decks of missionary verandas and colonial mansions, Malinowski sought to participate in the activities of the Trobriand people he studied. This—his—new brand of Participant Observation required a research period of at least one seasonal cycle, learning and practicing the native language, and eventually becoming an acknowedged member of the studied community. Questions about the living conditions *of* other people would thus be answered by means of the researcher’s experience *with* those people.^6^

This new understanding of fieldwork is committed, at least in theory, to inductive interpretation and theory building. It demands an attentive shift induced by contextual knowledge about other people’s lifeworlds. Crucially, the turn to participatory research acknowedges that attention is culturally coded and has to be redirected in the field. Participant Observation interlocks two distinct types of knowledge acquisition. Mere observation, on the one hand, is steered by attention and generates observational knowedge. At least in part, our observational habits tacitly guide our attention to what we typically observe, or to the unusual. Either way, what we end up observing is affected by our culturally shaped habits about where to look, and what to look *for*.

Participation, on the other hand, while guided by attention, *generates* it as well. As we join in a specific practice with others, we adjust our senses to what matters in that specific social setting of the practice. We learn from those who invite us to participate what is worth observing. In this sense, observation is not opposed to participation, but bound up with it.^7^ Ideally, participation and observation inform each other’s epistemological accomplishments. Together, they are channeled by the anthropologist as complementary tools to adapt their perceptual and corporeal habits and dispositions to local processes of perceiving and doing things. The aim is attentive immersion; a gradual adjustment of the senses mediated by Participant Observation (Bloom-Christen 2023a, 71).

Framing Participant Observation as a method that interlaces different levels of attention gives us a first set of clues for what kind of joint attention anthroplogists are aiming for. While ‘joint attention’ fails to surface as a technical term in the history of the development of social anthropology, it is easy to see how its actualisation is not only present, but critical: Joint attention signifies the shift from etic to emic interpretation of social meaning. In other words, joint attention drives the transition from the outsider perspective of a distant observer to an insider view of a community member.

But how and when, if at all, does the shift from solo to joint attention in ‘the field’ happen? Attentive immersion as defined above can be understood as an easy-access technique, a realistic goal, or an unattainable ideal. The next section will raise some problems with the adoption of a too low-threshhold notion of joint attention.

## Epistemological and Moral Concerns

Conceived as a route to knowledge, Participant Observation faces a set of serious epistemological, and by extension moral problems. The anthropolgist’s distinct positionality—their social and cultural background, their appearance and dispositions—makes the endeavor of producing social scientific knowledge through Participant Observation questionable, at the very least.^8^ The conundrum at the heart of these problems is of course more fundamental than the question whether one is similar enough to the people one seeks to perceptually join in order to access the notorious emic perspective. How can we share a perceptual field as a field of shared meaning at all? Can we jointly attend to something in the sense that we perceive the same thing *as that thing*?

For it appears that the kind of joint attention anthropologists strive towards is more demanding than what, for instance, John Campell had in mind with his example of two random people sharing the experience of watching a swan (Campbell 2005, 287). Knowingly perceiving the same entity together—in this case, a swan—is an example for what I will call a low-threshold form of joint attention where the greater contextual meaning and significance of the jointly perceived entity does not have to be shared. This weak form of joint attention does not require any common interest in the swan. How we each feel about that particular swan, about white birds in general, whether seeing it makes us sentimental or hungry, which memories it inspires or why we noticed the swan in the first place is irrelevant for this type of joint attention. All that matters is that we knowingly attend to the swan together.

The type of joint attention contemporary anthropologists are supposed to have in mind when embarking upon fieldwork is more demanding. The task is to access perceptual common knowledge of the kind Axel Seemann (2019) has proposed. Seemann frames perceptual common knowedge as a complex sociocognitive phenomenon emerging between joint agents and the perceived entity, each making up one corner of what he calls a triadic perceptual constellation (2019, 161–167). Adopting a Gricean model of communication,^9^ Seemann discusses how social agents achieve perceptual common knowledge through demonstrative reference in communication. His chapter on intention and communcation concludes by highlighting the importance of demonstrative indication of the location of the speaker’s intended reference. However, if the hearer “does not know where to look, demonstrative communication cannot get off the ground” (2019, 44). The complicated task of the anthropologist is to know which aspects of the shared environment are relevant for the greater web of meaning intended to be understood by means of Participant Observation. The overall mission is epistemologially more demanding because it requires knowledge of the broader system of meaning referenced by the studied social group, in order to then *know where to look*.

This is why a realtionalist account as defended by Campbell and Seemann are unsuitable to explain the demaning notion of joint attention aimed for in anthropological fieldwork.^10^ Such approaches envision an experiential relation to be sufficient for joint attention to arise. In the field, however, the triadic perceptual relation necessary for joint attention must be accompanied by a special kind of dyadic realtionship between the researcher and their research partners. In Felipe Léon’s terms of framing a non-reductive account of joint attention, it is vital that “co-attenders *relate* to one another as a ‘you’” (2021, 551, emphasis added). Expanding on this necessity to co-relate, the anthropologist must not merely recognise their research partner as a co-attender, but develop a sense of ‘you’ that includes contextual knowledge of this ‘you’’s lifeworld, thus engaging an idea of what matters to ‘you’.

One crucial condition for developing such a sense of ‘you’ is trust. In anthropological literature, trust is widely acknowledged as a societal resource that defines and shapes all spheres of everyday life, on an individual as well as an institutional level (Cohen and Sheringham 2016; Townley and Garfield 2013). The proposition that trust is key to found and foster ethically sound interactions is perhaps as well-established as the stern disciplinary belief in the revealing powers of participation itself. Trust is understood as an affective attitude that co-creates a sphere of acknowledging and sharing each other’s intentions—in other words, the sharing of what matters to us. Harking back to my own fieldwork experience, I recall a positive shift towards interpersonal trust after I broke my foot during the final period of my walking research. Entering public space with the visible vulnerability of my leg in a cast, I signalled trust towards my environment. This attitude was warmly received because it displayed positive expectations towards my social surrounding. I was taken to represent my research partners as trustworthy walking companions, thus making it more likely that they would respond with a similar affective attitude because I provided them with a normative reason to do so. Hans Bernhard Schmid (2013) has called the transformative feature of this joint affective display “the Power of Trust”, according to which trust is a partially self-fulfilling attitude (2013, 50–51).^11^

Incorporating this insight into the methodological framework of Participant Observation, we can postulate that interpersonal trust is fundamental for conducting fieldwork that is epistemologically valuable and morally sound. It is, however, not sufficient to bridge the gap of inequality regarding authority of knowledge production. For at some point—generally around the time when the fieldwork must come to an end because time and/or money is running out—the anthropologist will have to have reached the level of holistic understanding of how things hang together for the lifeworld she seeks to study that puts her in the epistemic position to make the sort of knowledge claims that are the core of ethnographic texts. Whether the criterion is met has to be decided on a case-to-case basis. And it is usually decided by the researcher alone.^12^ This creates an epistemological as well as a moral problem.

The epistemological problem is somewhat obvious: the outsider decides that she has become an insider without a clear protocol to check the probability or degree to which this is true. While it is now considered good practice to share and discuss one’s work with the studied community to see if its members agree with the researcher’s interpretation of their lives, the irreproducibility of Participant Observation makes it difficult to ascertain the emic accuracy of the anthropologist’s interpretation. And while more room has been given to original voices of research partners in recent decades, granting them co-authorship and acknowledgement as collaborators rather than mere informants (Fluehr-Lobban 2008; Collins et al. 2017), the conceptual framing of the research project, and thus its interpretive predominance, typically remains in the hand of the anthropologist.

The second—moral—problem follows from the first. The claim to joint attention is a claim not only about the lifeworlds of others, but one that shapes the discourse about the people described. Ethnograpies have scientific authority, influence policy-making and sometimes even make it into public discourse in the form of trade books.

The moral dimension of the problem begins with the acknowledgement of social asymmetry. Ethnographies evoke partial views—or, as Clifford and Marcus put it, “partial truths” (1986, 6). The statement can be read as a critical reminder of who is choosing the angle, making the cuts, and framing the picture. Participant Observation is often conducted in situations informed by social, cultural, and economic inequality (Abu-Lughod 1996). The “first world” investigates the “second” and the “third”, giving narrative authority to Western interpretations of social meanings. This raises the problem of how joint attention in the strong sense evoked above can emerge despite the often enormous cultural and social inequalities between the participants and the researcher. One way to put this problem is classically Foucauldian: in the study of human beings, the goals of power and the goals of knowledge cannot be separated.^13^ Our academic system of knowledge production feeds back into what we think is true and important—what we *ought* to pay attention to—which in turn reshapes our research focus. Power relations are present in any social situation. In anthropology, however, the stakes are especially high because the one-sided interpretation feeds into the discourse about how the described people make sense of the world, of how they live.

Anthropologists are in a powerful position of interpreting other people’s ways of life for a broader audience. What exactly they determine as participation matters—not only for their specialized scientific community, but for the greater narratives about cultures and peoples that are spun out of ethnographies bearing the authoritative stamp of recognized and validated academic work. It is therefore critical for anthropological theory and methodology to be clear about what joint attention is, and how to achieve it in a cross-cultural context.^14^

Embracing the method of Participant Observation, the prevalent assumption in anthropology is that jointly attending to other people’s lifeworlds is achievable, despite vast differences in contextual knowledge and experience. But what if joint attention, in its most demanding form, is not an achievable phenomenon, but an ideal never to be confirmed in its full-blown version?

## Towards Joint Attention: Relational Attunement and the Asymptotic Curve

Let us take a closer look at the perceptual timeline of Participant Observation with the example of my own fieldwork in Makhanda, a small town in the Eastern Cape Province of South Africa. Overall, I spent 13 months in the field, with a project on how the everyday use and accessablility of public space has been renegotiated since the end of apartheid in 1994. The main way I spent time with my research partners was walking together.^15^ Following the methodological framing of Participant Observation as summarised above, I expected that joint attention would guide me from the very beginning. The shared immersion in a perceptual field promised a shared view not only of the landscape ahead, but of its context-specific significance. But the relational attunement to what mattered to my research partners only grew slowly, bit by bit.^16^ An initial sense of a partially shared world developed after approximately 9 months, as I began to see not only what mattered to them, but why.

A vignette from my fieldwork will provide a more concrete idea of this process.

In Makhanda, everything slowed down for me. Walking to university, buying coffee, waiting for my research partners: it all became a task with a felt potential duration of forever. My hitherto lifeworld was streamlined and efficient. My fieldsite suffered from blatant socio-economic disparities and low levels of interracial social cohesion, which entailed manyfold decelerating consequences for the flow in public space. The difference between my habitual walking pace and the slower rhythm of Makhanda was imposing; it helped to be an observant outsider at first. The rupture I encountered had its own revealing quality that resided in the felt difference itself.^17^

During this period of adaption, I began to notice subtle nuances of how Makhandians were crossing paths in public. As I found myself developing a walking habit closer to the local tempo, I was often thwarted by Black pedestrians who not only entertained a slower pace, but also tended to walk in groups that would have to be circumvented or waited behind. As I began to partake in group walks with my Black research partners in the city center, I regularly noticed white pedestrians like myself trying to pass us by. Sometimes, they would give us looks of sheepish annoyance, or else utter the very (South African) British “sorry” as they stepped into the street, overtaking us who were clogging their path.

After such group walks I habitually assumed that walking slowly was a sign of indifference and in this sense, of a lack of clearly directed intention with regards to that specific joint action. Reminded of the findings from *Die Arbeitslosen von Marienthal* (Jahoda et al. 1975), which examined the sociography of an unemployed community, I asserted that lack of opportunity to find paid work causes the majority of (mostly Black) Makhandians to walk significantly slower compared to their (mostly white) employed fellow citicens.^18^ I suspected a vicious cycle between reduced opportunities and reduced aspiration.

Yet in the course of joining my research partners’ daily walks over an extended period of time, I became more attentive to what they saw and started to notice subtle hints that suggested a very different interpretation. A tempered gait, it dawned on me, could just as well be the expression of a very clear-cut intention. Crucially, it could be an expression of resilience against an abiding power structure.^19^ Intensifying the feeling of hierarchical segregation, the walking pace in the center of town was driven by the pace of white people—although today, the post office and the local bank branch located there serve not only the 8% of whites but also the remaining 92%. When walking through High Street, the town’s center lane with its colonial style buildings, the city’s aestetics appeared predominantly white, and the flow of bodily movement was faster than in the township. Whites hop out of their cars in front of restaurants and shops, where they consume goods while young Black men wait to guard the cars for a few Rand, rain or shine. White shoppers jump back into their mobile private safe spaces in public, dominating the government-financed roads supposedly built for everyone.

As a pedestrian, one has to be creative and claim public space differently. I started noticing that some of my research partners would cross the streets in an interesting way. Rather than looking at the approaching traffic, they would make a point of looking elsewhere: up; down; at me, their walking companion. By tilting their heads away from approaching traffic as they stepped into the street, they would signal that they do not see the approaching car, thus forcing the driver to stop. At first, I thought this to be another unintentional expression of inattentiveness, and urged my walking partners to look out for approaching vehicles instead of turning their gaze towards me. Later, however, I realized that this was in fact an effective (albeit risky) technique of crossing the street without having to wait for a driver to kindly stop.

As more research time passed, the sedate walking rhythm of my walking companions began to appear to me as an act of resistance against white dominance of public space, expressing itself in a physical rushing by, signaling who has “things to do”. My research partners were not giving into this normative notion of time-efficient commuting, but in their walking performed resistance against the white rhythm of busyness. Walking slowly meant openly breaking with the norms of local whites, whose strategy often was to rush through public space so as not to become a target for begging and mugging. While I had received the advice—from white person to white person—to never amble and always appear as if I was heading somewhere specific, a Black research partner pointed out to me that *if they want to get you, they will come and get you. If they want to pass*—she was now switching to talk about white people*—let them find their own way*. She was not going to make her everyday life be determined by white people’s stresses. Like many others I walked with in Makhanda, she was intentionally performing an act that ran counter to the speed forced upon her in midtown.

In time, this collection of experiences began to weave itself into a new, more holistic and contextualized picture of movement; one that took into account not only how people moved but, crucially, *why* they did so. This, in turn, sharpened my gaze and made me more attentive towards the manifold *hows*. I was now ready to perceive, for instance, my walking partner’s subtle gaze away from a passing white pedestrian or a slight deceleration, because I knew what it all was *for*. This knowledge not only turned me into a more attentive observer, but made me a better, complicit walking participant because I was now able to intentionally support the collective aim. Only once *this* was recognized—that is, once *participant attention* was knowingly established—I was openly and propositionally let in on “the secret”: *Let them wait.* Beginning to share the perceptual field of my walking companions, I would now notice movement guided by the overarching intention to reclaim ground and renegotiate power relations.

Bodily participation enabled me to open my perceptual field to this experience that imposed itself in the experiential transit of my gradual learning. With time I benefited from the above-described boost of contextual knowledge, which in turn afforded me deeper insights into the significance of my walking companions’ everyday movements. They were now, so to speak, sharing their attentive gaze with me. And I now knew *where to look*.^20^

After many months of living in Makhanda, I was invited to be a small part of the resistance. Walking slowly was a strategy to make those who were self-importantly rushing around feel their lives being put on halt, their plans thwarted, even if just for a moment. Feeling both the reluctance and willfulness that fueled the movement of walking allowed for an understanding of how the political sphere reaches into everyday habits and the bodily realm of approaching public space. Walking together, in this context, meant understanding, jointly attending, and rising to meet entrenched forces of oppression. In this sense, joint action offered a practicing platform to recalibrate my habits of attention. With time I began to anticipate the tacit realms of my research partners’ everday experiences, how movements reflected their preferences, how their attention was directed towards what contextually mattered to them. Joint attention in the strong sense was not simply and immediately attainable by following their ostensive gestures and listening to their explanations. Rather, there first needed to be in place some basic understanding of what to look for, which in turn had to be established in the context of a specific field site; cut out by me, embedded in their lives, slowly getting into view *what matters to them*.^21^

It is worth pausing to spell out the difference between joint attention in an everyday setting and joint attention as part of Participant Observation in the field. The latter is not essentially different from what transpires in shared intentional actions in everyday life. Building a house together, making breakfast together or walking together are all examples of shared practices—all of which to some degree require joint attention—that anthropologists may co-create as a part of their Participant Observations. The line between friendship and professionalism, between leisure and work, is easily blurred. Researchers may try to be ordinary participants in the daily lives of others, so there is no qualitative distinction to be made between ‘going for a walk with a research partner’ and ‘going for a walk with a friend’ with regard to the possibility, or the conditions of possibility, for the kind of joint action that leads to perceptual common knowledge. Yet the additional work of ethnographers is to subsequently put their experience into words that are relevant to the scientific community—which is itself its own, overarching goal not shared with their research partners. In this respect, the anthropologist is a special agent. Her professional goal is not to help build the house, but to understand what building that house means to the co-builder.

Let us insert this special agent into the above-discussed account offered by Seemann, who opens his book *The Shared World* (2019) with the story of the raft of the Medusa. 15 shipwreck survivors, huddled together on a raft, end up jointly attending to an approaching ship which is supposed to safe their lives. In this scenario, joint attention is tighly bound up with a shared world the castaways inhabit—the raft, the sea, the ship in sight. They share a story that began before the rescue ship emerges on the horizon. Now, if we were to put an anthropologist on the raft just as the saving ship comes into view, she would need a minute to catch up in order to understand the contextual significance of what lies ahead. To some degree, it is always like this when entering a new fieldsite: the story begins in medias res.

Returning to the example of my own fieldwork experience, my research partners and I jointly established a shared sphere of participant attention, which took time to build and started with solitary observation from my side.^22^ The perceptual timeline of joint attention in Participant Observation looks as follows:

$${\text{Observation}} \to {\text{Participant Observation}} \to {\text{Joint Attention}} \to {\text{Participant Attention}}$$

Participation begins with attention as a form of distant observation, which merges from participant observation into joint attention, which in turn allows for participant attention. Once this epistemological stage is reached, further observation will be contextually informed and attentively targeted more aptly, allowing for a new hermeneutical cycle of understanding to begin:
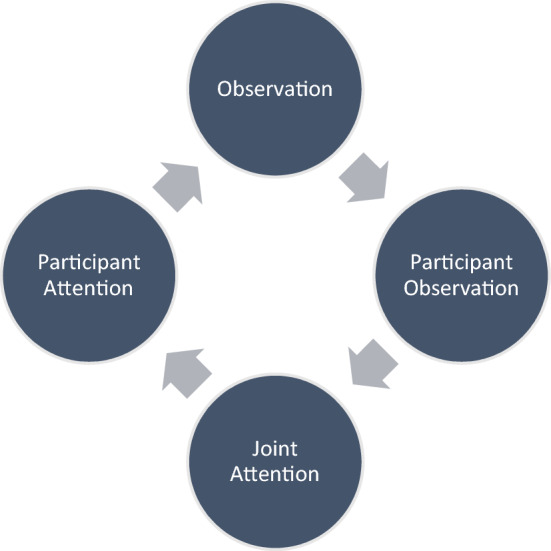


By moving joint attention from the beginning to the middle section of fieldwork practice, we allow for the backgound context to emerge in order to then jointly attend to the world as *one world*.^23^

What does this mean for the philosophical debate on what joint attention is? I propose that exploring the conditions of joint attention as a hard-to-reach goal in Participant Observation enriches the philosophical study of the phenomenon as a shared practice. This exploration does not merely serve to shine a light back on anthropology itself, but also as a stepping stone to understand the epistemological conditions under which joint attention emerges. On this high-threshold account, the triadic nature of joint attention is dependent on the presence of a high-demand dyadic relationship between the jointly attentive subjects that represents the other as a ‘you’.

This high-threshold form of joint attention should be seen as part of a spectrum from weak to strong forms of joint attention, with the acknowledgement of the curve towards sharedness as asymptotic. That is, the acknowledgement that there cannot be such thing as a full overlap in contextual meaning, but at best asymptotic approximation.

To clarify this final point, it is worth inspecting the metaphors offered to illuminate the nature and function of attention. Philosophical attempts to define attention have been diverse and rich. Attention has been described as a gatekeeper to consciousness (Wu 2014), the act of perceptual highlighting (Campbell 2002) or as a means to structure mental states according to priorities (Watzl 2017). Alfred Schütz, whose work on perception and relevance has been vital for phenomenological anthropology, describes attention as a special kind of theme-picking (1982). To attend to something together, on this account, means to pick the theme together, to then knowingly share a *Blickstrahl* (beam gaze).^24^ In the scenario of watching a swan together with Campbell, phenomenological anthropology highlights the significance of the web of meaning in which the “reality accents”, as Schütz has it, are set. Drawing on Husserl’s observation that some aspects in our lives are relevant—that is, thematic—while others recede into the horizon, Schütz distinguishes three kinds of relevance: *Topical relevance* makes the unfamiliar problematic as something that stands out (1982, 56–66). In fieldwork, this would translate to the kind of attention most often occurring during the initial research period guided by observation. With *interpretational relevance* Schütz puts into focus that mental representations need interpretation in order to be placed in a system of relevance (1982, 67–78). This aspect of attention delineates the period of fieldwork where contextual knowledge has been gathered and interpretations are considered in the light of the given context. Finally, *motivational relevance* targets the decision to act upon what lays before us (1982, 78–86). Joint attention as it occurs in joint action in the field becomes the guiding motivational force for participation in the light of knowing what matters to the other.

The notion of ‘the other’ featuring in the title of this article finally leads us to Schütz’s work on the stranger (*Der Fremde*, 1972), as it is in this text Schütz applies his view of attention as theme-picking to the phenomenon of assimilation and perceptual approximation. In his concluding remarks on the process of social assimilation, he describes how the stranger can shed his strangeness, namely by examining the cultural patterns of the foreign group until he has internalized them as a self-evident (Schütz 1972, 64–65).

Within the framework of Participant Observation, our special agent, the anthropologist, starts out as the stranger by choice. The Schützian assimilation process can perhaps be illustrated with the image of peeling a lettuce. Layer by layer, the stranger discovers deeper levels of contextual relevance in her new environment, the peeling of each layer resembling individual assimilation steps. The removal of the last layer is possibly no longer even felt as such—Schütz calls this *Denken-wie-üblich*—thinking as usual (1972, 58). And inside there is nothing; the stranger has ceased to be.

This metaphor is not meant to portray actual assimilation, but to schematically convey an ideal. It is this ideal that finds expression in the high-threshold notion of joint attention proceeding in the light of *fully* knowing what matter so the other. The *good* anthropologist, rather than assuming to have appropriately set her “reality accents” in her research proposal, is aware of the asymptotic nature towards unfamiliar cultural patterns. Against the backdrop of individual, cultural and social background, participant attention becomes the ultimate goal of joint action.

## Conclusion

To understand the conditions and functions of joint attention is a core question for both philosophy of perception and anthropological fieldword. Both fields of inquiry, although with different focal points and little conceptual consensus, seek to provide theoretical tools and empirical insights for the phenomenon of joint attention and the shapes it takes in human everyday life. The aim of this article was to translate and connect those two strands of discourse to allow each of them to benefit from the other’s findings.

Participant Observation presupposes a shared awareness of what matters to the context in which the shared practice takes place. This paper argued that joint attention is an interim goal rather than a necessary precondition for epistemologically valid Participant Observation. Joint attention, as pursued in anthropological fieldwork, is the gateway into the zone of an epistemic position from where social scientific knowledge on the basis of Particpant Observation can be gained. The anthropological sense of joint attention was thus discussed as a high-threshold version of the phenomenon.

I first introduced the emergence and growing importance of Participant Observation in the disciplinary history of anthropology, identifying a demanding notion of joint attention in the discipline’s methodological toolkit. I then showed how Participant Observation is a problematic, yet potentially uniquely fruitful way to gain contextual knowledge about the life circumstances of other human beings. By moving joint attention from the beginning to the middle part of a fieldwork period, I argued that it is both a source and a precursor of social scientific knowledge in anthropology. The phenomenological tradition finds expression for this exact moment, the ideal of intuitive access to another’s mind: “I “understand” you without necessarily paying any attention to the acts of understanding themselves. This is because, since I live in the same world as you, I live *in* the acts of understanding you” (Schütz 1967, 149). By means of attentive participation, the anthropologist strives towards joint attention which in turn will, eventually, gradually, and likely only partially or momentarily, allow her to live in the acts of understanding the other.^25^

Merging two intertwined yet distinct modes of knowledge acquisition—observation and participation—Participant Observation augurs access to local meanings of practices that reflect people’s self-understanding of their everyday lives. One necessary precondition for reaching this state is a somewhat shared lifeworld. The anthropologist is in the special position to be in the business of moving into unfamiliar social settings or, to repeat the *SAGE Handbook of Social Anthropology* definition of Participant Observation, to live “in a culture that is not your own while also keeping a detailed record of your observations” (2012, xii). The boundaries between one’s own attentive habits and those practiced by people in different cultures are blurred, but the emphasis of cross-cultural research makes anthropology a resourceful ally for philosophical, empirically informed research on joint attention. In this way, Participant Observation illuminates the structure of joint attention which, in turn, provides novel perspectives onto the understanding of mental events shaped by shared perception.

There is an additional insight to be gained from asking about understanding that comes from acting together beyond the point of paying attention to the specific shared practice. Schütz is unfolding the idea of an ideal understanding of the other by virtue of co-acting in the world they live in. Once this ideal is reached, no more attention to the act of understanding itself is necessary as you and I intuitively act together in our shared world.

In order to address the epistemological as well as the moral issues arising with knowledge claims based on Participant Observation, the anthropologist, while constantly *en route* towards that ideal, must keep in mind the asymptotic nature of her epistemic endeavor. In addition, research partners should be considered veritable collaborators and potential co-authors. In this respect, anthropology still has a long way to go. While progress has been made towards more inclusive fieldwork practices and ethnographic co-authorship by feminist anthropologists,^26^ the norm is still to write *about*, not *with* others. In this sense, the high-threshold account of joint attention outlined here could be applied not only to better understand the lifeworlds of others *outside* the scientific community, but also to become more attentive towards the experiences of minority groups within the borders of academia.

## Footnotes

1 Asking versions of this question, anthropologists have advanced the idea of cultural variation in how attention actualizes (Duranti 2015) and the role attention plays in social interactions across different cultures (Masuda et al. 2020). Especially phenomenologically minded scholars have taken up the notion of different ways of perceiving the world and by extension, different ways of paying attention. Race, gender, class, and their lived intersections (Fanon 1952, Young 1980, Desjarlais 1997, Ahmed 2006, Ortega 2016, Desjarlais and Throop 2011) are the main categories in which we can find a growing body of ethnographically informed literature on what people pay attention to, and why.

2 An example for fieldwork on attention, albeit from psychology, is Barbara Rogoff’s work on cultural variation in attention in children. The key idea, tested on children from different cultural backgrounds, is that cultures vary in how much purposeful joint attention matters for learning. See Chavajay and Rogoff (1999) and Silva et al. (2015).

3 Throughout this article, ‘Participant Observation’ discussed as a method-label is capitalized as a proper noun. ‘Participation’ and ‘observation’ examined as separate activities are spelt as common nouns.

4 While all senses are involved when in the field, the perceptual paradigm of how to pay attention—that is, how to be a participant observer—is visual.

5 The further historical development of anthropology as a scientific discipline is accompanied by the positivist endorsement of testing hypotheses. By predetermining what to observe before entering the field, this school of anthropologists followed the idea that fieldwork requires the researcher to first determine what to observe, to then follow through with it in order to confirm, relativize or falsify the initial assumptions. The belief that only observational evidence that is rationalized with the tools of logic can be a source of scientific knowledge affected anthropology in the sense that it became normal to think of observation as a method in its own right. The underlying presumption was that observation can be practiced objectively, and that the social world can be analyzed and dissected in the same way that the natural sciences conduct experiments.

6 I am here paraphrasing the ideal research technique as described by Malinowski himself. Infamously, his posthumously published private field diaries reveal a conflicted, hostile, at times deprecatory attitude towards his research partners (Malinowski 1989). For a concise account of Malinowski’s “ethnographic self-fashioning”, see Clifford (1988), 92–113. For a set of challenges posed to Participant Observation, see Bloom-Christen (2023a), 63–65.

7 This is not to say that participation and observation are always in balance or equally in play. Indeed, normally, one tends to prevail over the other.

8 The anthropological literature on knowledge, truth, and interpretation is extensive and detailed. For an overview, see the collected volume “Knowing how to know: Fieldwork and the ethnographic present” (Halstead, Hirsch and Okely 2008). For an in-depth ethnographic account of truth as a matter of cultural, epistemological, and ontological difference, see Martin Holbraad’s 2012 book “Truth in Motion: The Recursive Anthropology of Cuban Divination”.

9 A Gricean model of communication works on an intention-based semantics and is commonly framed as inferential. Importantly, Seemann abandons the Gricean intentional model later in his book, siding with Campbell that it “does not provide a suitable basis of thinking about the foundations of communication” (2019, 198n4).

10 This includes lean versions of relationalism such as presented by Campbell (2005), merely attesting an extensional triadic relationship between two subjects and an object of their attention, as well as rich versions of realtionalism as proposed by Wilby (2023), where in addition to this triadic relationship, joint attention “must embody a conceptual-like shape or structure that allows it to act as an epistemically significant interface between oneself, one’s co-attender, and the world” (2023, 135).

11 Schmid (2013) elaborates in detail on how interpersonal trust comes into being as a partially self-fulfilling attitude. Schmid’s view differs from instrumentalist conceptions of trust as a bet on others’ future behaviour (as found in Hardin 2006). Rather, “the Power of Trust” stands for attitudes towards the future that help fulfil its desired becoming. For a thorough, complementary examination of the characteristics of affective attitudes, see Jones (1996).

12 However, there is notable history of anthropologists questioning whether others have met the criterions of Participant Observation and whether they draw likely conclusions. Two famous examples are the Freeman-Mead debate about the accuracy of Margaret Mead’s fieldwork findings in Samoa (see Shankman 2009 for an overview) and the case ‘Obeyesekere against Sahlins’ (Obeyesekere 1997; Sahlins 1995) about whether native Hawaiians believed the British explorer James Cook to be a deity. These debates mark deeper problems in anthropology, such as the question whether Western scholars are in the epistemic position to understand non-Western cultures at all.

13 This point is only at the fringe of a much larger debate on authority, knowledge production and the sociology of knowledge in general. Foucault calls this intertwinement “power/knowledge”, since it combines into a unified whole “the deployment of force and the establishment of truth” (Foucault 1975, 184).

14 While this article’s aim is to highlight the productive role of joint attention in Participant Observation and to explore avenues to enhance the method, it is important to flag the limitations of this account. As argued above, joint attention should be seen as an ideal. However, raising the ‘epistemic bar’ is not the only, nor necessarily the best way to improve ethical standards in anthropological fieldwork. The inherent limitations to the method which is, by definition, dominated by the experience of the researcher rather than their research partners, should be seen as an urgent invitation to seek for alternative modes of participatory research. The bigger, underlying problems are deeply rooted in the history of anthropology itself. Critically discussing cardinal issues of anthropology from a philosophical standpoint, Michael Lambek has recently noted in his Tanner Lecture that “one of the missions of anthropology” is “pursuing philosophy with *other* people in it” (Lambek 2021, 7). In other words, anthropology remains caught up in a self-understanding of relating to ‘the other’ and reporting on that relation rather than offering others a platform to give an emic account themselves.

15 This research was conducted in the context of my PhD project “Walking Together: Perils of Participation in Anthropology and Philosophy of Action” (2023b). My research partners were offered and have explicitly declined the option for co-authorship.

16 I borrow the term ‘relational attunement’ from clinical psychology, describing the learning process of anticipating the thoughts and desires of others. See Erskine 1998.

17 In “Knowing the City” (2023c), I discuss in more detail the epistemic benefits of embodied experiences of rupture when walking in unfamiliar urban spaces.

18 Today considered a classic in the literature of social stratification, this multimethodological study explores the impact of job loss and long-term unemployment for a town community of approximately 800 people in 1930 Austria. A whole community was laid off at the same time as a large textile factory in the village of Marienthal was shut down during a depression. The study concluded that continuing unemployment leads to a state of apathy in which the unemployed cease to make use of even the little opportunity they have left.

19 One of many remnants of South Africa’s racist past, the informal “zoning” of public space into different walking speed zones brings to mind the Group Areas Act of 1950, which mandated residential and business zones in cities for each racial group. Other races could neither live in nor own land in those areas. In effect, by the late 1950s, more than 80 percent of South Africa’s land was owned by whites, and people defined as non-whites had to carry documents allowing them to enter restricted areas. Though that policy has been abandoned, its impact lingers on.

20 In practice, this manifested itself for example in theim holding my arm as we walked towards or in front of a swiftly approaching white person, externally signaling our togetherness as a walking unit. Well-aware that we were thereby producing a walking barricade, they were now letting me join in their practice.

21 The claim that it takes time to understand the complexity of foreign social settings is itself not controversial, but rather self-evident. The larger point behind the spelling out of the learning curve within fieldwork, however, is that it is the difficulty of (mis)understanding itself that distinguishes Participant Observation as a worthwhile research method. Rather than seeing the process of learning merely as an unavoidable preparatory factor that is followed by a time of gathering information “in the light” of sufficient contextual knowledge, the period of getting things wrong is an integral part of the understanding itself. This is because attention emerges also where habits are disrupted, where things go wrong, and alleged understanding turns out to be misunderstanding. Disrupting one’s own attentive habits, then, is not a precursor, but a component of ethnographic knowledge (see Bloom-Christen 2023b).

22 This tracks with findings from within neuroscience and linguistic anthropology. Several studies on learning provide evidence suggesting that prior knowledge of context of a particular practice enables subsequent embedded meaning to be detected, comprehended, and remembered better (Bransford and Johnson 1972). In their book on relevance, Sperber and Wilson (1986) propose that communication is possible because of shared background knowledge and joint attention to the relevant background knowledge needed to mutually understand what is being communicated. As Raykov et al. (2022) confirm, sharing contextual knowledge strengthens inter-subject synchrony in the semantic network. Their findings also support the claim that contextual prior knowledge constrains how new information is interpreted. (2022, 17).

23 For a comprehensive summary on the ontological turn, see Holbraad and Pedersen 2017. For an argument in favor of a “One World Anthropology”, see Ingold 2018.

24 The literal *Blickstrahl* signifying visual attention is traceable by neuroimaging techniques such as electroencephalograph (EEG) or video recording of eye movement. While such research seems apt for understanding behavioral dimensions of attentive agency (e.g., Nielbo and Sørensen 2016), the broader framework of agent *intentions* remains a black box. Anthropology seeks to open this box by considering the wider context of peoples’ life circumstances and their motivation for attentive agency in it.

25 Schütz’s point about living in the act of understanding the other picks up on everyday notions of the gradual “automatization” of learning to do things. Examples include playing the guitar, tennis, or riding a bike; at first one needs to consciously pay attention to certain things one is trying to learn, but eventually one simply does it without thinking about it.

26 See for instance Abu-Lughod (1990), Visweswaran (1994), Abu-Lughod (1996), Stewart (2007), Bloom-Christen and Grunow (2022).
